# The Residual ISI for Which the Convolutional Noise Probability Density Function Associated with the Blind Adaptive Deconvolution Problem Turns Approximately Gaussian

**DOI:** 10.3390/e24070989

**Published:** 2022-07-17

**Authors:** Monika Pinchas

**Affiliations:** Department of Electrical and Electronic Engineering, Ariel University, Ariel 40700, Israel; monikap@ariel.ac.il

**Keywords:** residual ISI, MET, moment truncation technique, Laplace’s integral method, blind adaptive deconvolution, Lagrange multipliers

## Abstract

In a blind adaptive deconvolution problem, the convolutional noise observed at the output of the deconvolution process, in addition to the required source signal, is—according to the literature—assumed to be a Gaussian process when the deconvolution process (the blind adaptive equalizer) is deep in its convergence state. Namely, when the convolutional noise sequence or, equivalently, the residual inter-symbol interference (ISI) is considered small. Up to now, no closed-form approximated expression is given for the residual ISI, where the Gaussian model can be used to describe the convolutional noise probability density function (pdf). In this paper, we use the Maximum Entropy density technique, Lagrange’s Integral method, and quasi-moment truncation technique to obtain an approximated closed-form equation for the residual ISI where the Gaussian model can be used to approximately describe the convolutional noise pdf. We will show, based on this approximated closed-form equation for the residual ISI, that the Gaussian model can be used to approximately describe the convolutional noise pdf just before the equalizer has converged, even at a residual ISI level where the “eye diagram” is still very closed, namely, where the residual ISI can not be considered as small.

## 1. Introduction

The convolutional noise brought on by a blind adaptive deconvolution or blind adaptive equalization system is the subject of this research. In a blind adaptive deconvolution (blind adaptive equalization) system, all that is available is the output sequence of an unidentified linear system (channel), and the goal is to recover the input sequence of that system without the aid of a training sequence [1,2,3,4,5,6,7]. Numerous fields, including seismology, underwater acoustics, image restoration, and digital communication, use blind adaptive deconvolution systems [7,8,9,10,11,12,13,14,15,16,17,18,19,20,21,22,23,24,25,26,27,28,29,30,31,32,33,34,35,36,37,38,39]. For a moment, let us think about the scenario of digital communication, where a source signal gets convolutedly distorted during transmission between its symbols and the channel impulse response. The recovery process is significantly hampered by this distortion, known as ISI, which produces detrimental distortions [39]. An adaptive blind equalizer is used to solve the ISI problem. The coefficients of the ideal blind adaptive equalizer are unknown because the channel coefficients are unknown. In a blind adaptive deconvolution system, the equalizer’s coefficients are only approximated values to the optimal ones, resulting in the addition of an error signal to the source signal at the deconvolution process’s output. This error signal is defined as the convolutional noise and is closely related to the residual ISI. If the system’s residual ISI level is relatively low after the blind adaptive equalization, it means that the convolutional noise is considered very small. Until recently, the Gaussian pdf was frequently used [1,2,6,34,40,41,42,43] to approximate the convolutional noise pdf throughout the iterative deconvolution process. However, according to [41], the convolutional noise pdf tends approximately to a Gaussian pdf only at the end of the iterative deconvolution process when the equalizer has converged to a relatively low residual ISI (where the convolutional noise is relatively low). The input sequence and the convolutional noise sequence are significantly associated in the early stages of the iterative deconvolution process because the ISI is often high, and the convolutional noise pdf is more uniform than Gaussian [41,44]. It should be noted that even though the Gaussian model was utilized for the convolutional noise sequence throughout the entire deconvolution procedure in [1,2,6,34,40,41,42,43], satisfying equalization performances were obtained. Recently, an attempt was made to approximate the convolutional noise pdf differently than with the Gaussian one in order to obtain improved equalization performance. In [4], the maximum entropy density approximation method [1,2,45,46] and Lagrange multipliers up to order four were used to approximate the convolutional noise pdf. In [5], the convolutional noise pdf was approximated with the Edgeworth Expansion series [47,48] up to order six. In [3], the Generalized Gaussian Density (GGD) [49,50] function and the Edgeworth Expansion [47,48] were applied to approximate the convolutional noise pdf. The GGD [49,50] is based on a shape parameter that modifies the pdf, which for shape parameters equal to one, two, and infinity, respectively, may have a Laplacian, or double exponential distribution, a Gaussian distribution, or a uniform distribution. Even though equalization performance was enhanced with these new approximation techniques for the convolutional noise pdf compared with the Gaussian case, a much higher equalization performance improvement was expected but not achieved. Thus, it makes us wonder if maybe the Gaussian model for the convolutional noise pdf is approximately correct even when the residual ISI is not so small. There is currently no closed-form approximated expression for the residual ISI where the Gaussian model can be used to describe approximately the convolutional noise pdf. It is well known that the equalizer can converge at a residual ISI level that might not be very low even high, depending on the applied step-size parameter used in the equalizer’s coefficients update mechanism. Furthermore, the equalization performance from the residual ISI point of view depends on the chosen equalization algorithm, equalizer’s tap length, input signal statistics, channel characteristics, and step size parameter. Thus, any closed-form approximated expression for the residual ISI where the convolutional noise pdf can be considered approximately Gaussian, must be a function of all the abovementioned parameters playing a role in the equalization performance from the residual ISI point of view. In this study, we address the noiseless and 16 Quadrature Amplitude Modulation (16QAM) situation. We use the Maximum Entropy density approximation technique [1,2,45,46] with Lagrange multipliers up to order six to approximate the pdf of the real part of the convolutional noise pdf. Then, we use Laplace’s integral method [51] and quasi-moment truncation technique [48] in order to obtain an approximated closed-form expression for the residual ISI for which the pdf of the real part of the convolutional noise can be approximately considered as Gaussian. This closed-form approximated expression is a function of the channel power, input sequence statistics, equalizer’s tap length and properties of the chosen equalizer. It is appropriate for the type of blind adaptive equalizers where the error that is given into the adaptive mechanism that updates the equalizer’s taps can be described as a polynomial function of the equalized output up to order three. It should be pointed out that Godard’s algorithm [52], for example, belongs to the mentioned type of blind equalizers. Based on this closed-form approximated expression for the residual ISI, we are able to show via simulation results that the Gaussian assumption for the convolutional noise pdf can be approximately made just before the equalizer has converged, even at a residual ISI level where the “eye diagram” is still very closed, namely, where the residual ISI can not be considered as very small. At that level of residual ISI, the fourth Lagrange multiplier in the approximated pdf of the real part of the convolutional noise is approximately zero, while the sixth Lagrange multiplier is very small and tends approximately to zero. Please note, since we deal with a two-independent quadrature carrier input case, all the even Lagrange multipliers in the convolutional noise pdf approximation are zero.

## 2. System Description

**Figure 1:**
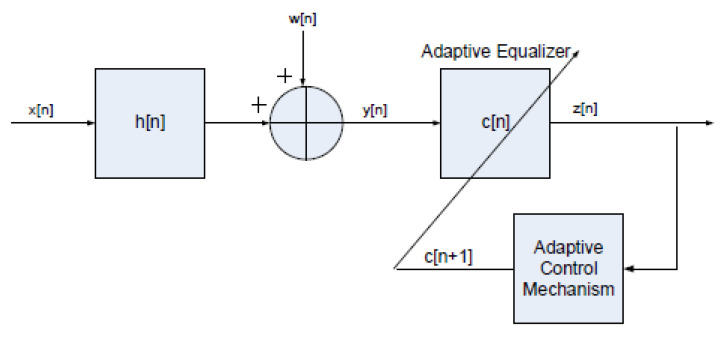
System description.

Figure 1 provides a description of the system under study. Please note that the described system is recalled from [53,54]. It should be noted here that the described system in Figure 1 is not unique but is a general description of a system using a blind adaptive equalizer to recover the input sequence from an unknown linear channel, as is the case in [52,55,56] and in other works.

In this paper, we make the following assumptions:

1. The input sequence x[n] is a **16QAM source**, which can be expressed as $x\left[n\right]=x_{1}\left[n\right]+jx_{2}\left[n\right]$ where $x_{1}\left[n\right]$ and $x_{2}\left[n\right]$ are x[n]’s real and imaginary parts, respectively. 16QAM is a modulation that uses ± {1,3} levels for in-phase and quadrature components. E[x[n]]=0 and E[(·)] denotes the expectation of (·). The real and imaginary parts of x[n] are independent.
2. The unidentified channel h[n] is a linear time-invariant filter that may not have a minimum phase and whose transfer function lacks “deep zeros,” or zeros that are sufficiently removed from the unit circle. The channel’s tap length is *R*.
3. The filter c[n] is a tap-delay line.
4. The channel noise w[n] is an additive Gaussian white noise.

The sequence x[n] is sent through the channel h[n] where the output sequence from the channel is corrupted with noise w[n]. The input sequence to the blind adaptive equalizer is denoted as y[n] and expressed by:

(1) y[n]=x[n]∗h[n]+w[n]

where the convolution operation is indicated by the symbol ∗. z[n] is the equalized output sequence and given by:

(2) $$\begin{array}{c} z[n]=c[n]*y[n]=x[n]*s[n]+c[n]*w[n]=\\ x[n]+p[n]+c[n]*w[n]\\ \mathrm{where}\\ s[n]=h[n]*c[n];\;\;p[n]=x[n]*(s[n]-\delta [n]) \end{array}$$

where p[n] is the convolutional noise arising for not having the optimal values for the equalizer’s coefficients. The real and imaginary components of p[n] are denoted in the following as $p_{1}\left[n\right]$ and $p_{2}\left[n\right]$, respectively. Since we deal with a two independent quadrature carrier constellation input, $E\left[x_{1}^{v}\left[n\right]\right]=E\left[x_{2}^{v}\left[n\right]\right]$ and $E\left[p_{1}^{v}\left[n\right]\right]=E\left[p_{2}^{v}\left[n\right]\right]$ for v=1,2,…,V. The ISI expression is used to evaluate the equalizer’s performance:

(3) $$ISI=\frac{\sum_{m}|s_{m}{\left[n\right]|}^{2}-{\left|s_{m}\left[n\right]\right|}_{max}^{2}}{|s_{m}{\left[n\right]|}_{max}^{2}}$$

where |·| is the value of (·) in absolute terms and $|s_{m}{\left[n\right]|}_{max}$ is the component of s[n] in (2) with the highest absolute value. For the noiseless case, $|s_{m}{\left[n\right]|}_{max}^{2}=1$ and the equalizer has entered its convergence state, we may write according to [1]:

(4) $$ISI≃10log_{10}\left(\frac{E[|p\left[n\right]{|}^{2}]}{E[|x\left[n\right]{|}^{2}]}\right)\;\mathrm{for}\;{\left|s_{m}\left[n\right]\right|}_{max}^{2}=1$$

Since we are dealing with the 16QAM constellation input situation of two independent quadrature carriers, we may express (4) as:

(5) $$ISI≃10log_{10}\left(\frac{E[p_{1}^{2}\left[n\right]]}{E[x_{1}^{2}\left[n\right]]}\right)\;\mathrm{for}\;{\left|s_{m}\left[n\right]\right|}_{max}^{2}=1$$

The equalizer’s update mechanism can be described by:

(6) $$\underline{c}\left[n+1\right]=\underline{c}\left[n\right]-\mu \frac{\partial F[n]}{\partial z[n]}{\underline{y}}^{*}\left[n\right]$$

where the conjugate operation is ()*, the step-size parameter is μ, the cost function is F[n], $\frac{\partial F[n]}{\partial z[n]}$ is the cost function’s derivation from the equalized output sequence and $\underline{c}\left[n\right]$ is the equalizer vector where $\underline{y}\left[n\right]={\left[y\left[n\right]\ldots y\left[n-N+1\right]\right]}^{T}$ is the input vector. The equalizer’s tap length is *N*, and the operator ()*^T^* stands for the transpose of the function (). In this paper, the MMA algorithm [55,56] and Godard’s algorithm [52] are used. The equalizer’s coefficients are updated according to the MMA algorithm ([55,56]) by:

(7) $$\begin{array}{c} \underline{c}\left[n+1\right]=\underline{c}\left[n\right]-\mu_{MMA}\left[Re\left[z\left[n\right]\right]\left[|Re\left[z\left[n\right]\right]{|}^{2}-\frac{E\left[|x_{1}{\left[n\right]|}^{4}\right]}{E\left[|x_{1}{\left[n\right]|}^{2}\right]}\right]+\right.\\ \left.j\left[Im\left[z\left[n\right]\right]\right]\left[|Im\left[z\left[n\right]\right]{|}^{2}-\frac{E\left[|x_{2}{\left[n\right]|}^{4}\right]}{E\left[|x_{2}{\left[n\right]|}^{2}\right]}\right]\right]{\underline{y}}^{*}\left[n\right] \end{array}$$

where $\mu_{MMA}\geq 0$, $Re\left[\cdot \right]$ and $Im\left[\cdot \right]$ are the real and imaginary parts of $\left[\cdot \right]$, respectively. The equalizer’s coefficients are updated according to Godard’s algorithm [52] by:

(8) $$\underline{c}\left[n+1\right]=\underline{c}\left[n\right]-\mu_{G}\left({\left|z[n]\right|}^{2}-\frac{E\left[{\left|x[n]\right|}^{4}\right]}{E\left[{\left|x[n]\right|}^{2}\right]}\right)z\left[n\right]$$

where $\mu_{G}\geq 0$. It needs to be stated that according to [57], Godard’s algorithm is one of the most widely used blind equalization algorithms and has become the workhorse for blind equalization. According to [57], Godard’s algorithm is carrier phase independent. Therefore, carrier synchronization is not necessary prior to blind equalization. However, an arbitrary phase rotation is present in the constellation visible at the equalized output sequence [57]. As a result, a phase rotator is necessary at the equalizer’s convergence state in order to spin the constellation back into place [57]. The MMA method ([55,56]) avoids the necessity for a phase rotator, in accordance with [57], because it employs a separate error-calculation, i.e., for the real and imaginary parts of the received signal, separately. In this paper, we assume that $\frac{\partial F[n]}{\partial z[n]}$ can be expressed as a polynomial function of the equalized output namely as:

(9) $$P\left[z\left[n\right]\right]=\frac{\partial F[n]}{\partial z[n]}$$

Thus, based on (9) and [58], the real part of the polynomial function P[z[n]] of order up to three can be expressed by:

(10) $$Re\left[P[z[n]]\right]=a_{1}z_{1}\left[n\right]+a_{3}z_{1}^{3}\left[n\right]+a_{12}z_{1}\left[n\right]z_{2}^{2}\left[n\right]$$

where the real and imaginary components of the equalized output z[n] are denoted as $z_{1}\left[n\right]$ and $z_{2}\left[n\right]$, respectively. Please note that for the 16QAM constellation input case, $a_{1}=-\frac{E\left[{\left|x[n]\right|}^{4}\right]}{E\left[{\left|x[n]\right|}^{2}\right]}$, $a_{3}=1$ and $a_{12}=1$ for Godard’s algorithm [52] while for the MMA algorithm ([55,56]) we have that $a_{1}=-\frac{E\left[{\left|x_{1}\left[n\right]\right|}^{4}\right]}{E\left[{\left|x_{1}\left[n\right]\right|}^{2}\right]}$, $a_{3}=1$ and $a_{12}=0$.

## 3. The Residual ISI That Leads Approximately to a Gaussian pdf for the Convolutional Noise

In this section, we present a closed-form approximated expression for the residual ISI as a function of the system’s parameters (step-size parameter, input constellation statistics, Equalizer’s tap length, channel power and properties of the chosen equalizer via $a_{1}$, $a_{3}$ and $a_{12}$) for which the convolutional noise pdf can be approximately considered as a Gaussian pdf.

**Theorem** **1.** *The residual ISI ($ISI_{res}$) for which the convolutional noise pdf associated with the blind adaptive equalization problem is approximately a Gaussian pdf can be expressed for the noiseless case by:*

(11) $$\begin{array}{c} ISI_{res}≃10log_{10}m_{p}-10log_{10}m_{2};\;\;m_{2}=E\left[x_{1}^{2}\left[n\right]\right];\;\;m_{p}=E\left[p_{1}{\left[n\right]}^{2}\right] \end{array}$$

*where $m_{p}$ is the solution of the following equation:*

(12) $$\begin{array}{c} A_{1}m_{p}^{3}+A_{2}m_{p}^{2}+A_{3}m_{p}+A_{4}=0\\ where\\ A_{1}=105T\left(3B^{2}a_{12}^{2}\left(\frac{3}{T}-1\right)+B^{2}a_{3}^{2}\left(\frac{135}{T}-15\right)+6B^{2}a_{3}a_{12}\left(\frac{3}{T}-1\right)\right)-\\ 30B^{2}\left(a_{3}^{2}\left(\frac{28\;350}{T}-945\right)-3a_{12}^{2}\left(\frac{3}{T}-1\right)\left(\frac{135}{T}-15\right)+2a_{3}a_{12}\left(\frac{1890}{T}-105\right)\right)\\ A_{2}=12a_{12}\left(\frac{135}{T}-15\right)-30B^{2}\left(6m_{2}a_{12}^{2}\left(\frac{135}{T}-15\right)+15a_{3}^{2}m_{2}\left(\frac{1890}{T}-105\right)-\right.\\ \left.9m_{2}a_{12}^{2}{\left(\frac{3}{T}-1\right)}^{2}+2a_{1}a_{12}\left(\frac{135}{T}-15\right)+2a_{1}a_{3}\left(\frac{1890}{T}-105\right)+\right.\\ \left.12a_{3}m_{2}a_{12}\left(\frac{135}{T}-15\right)+2a_{3}m_{2}a_{12}\left(\frac{1890}{T}-105\right)\right)+\\ 12a_{3}\left(\frac{1890}{T}-105\right)+105T\left(2B\left(a_{12}-3a_{3}\left(\frac{3}{T}-1\right)\right)-2B^{2}a_{1}a_{12}-\right.\\ \left.6B^{2}m_{2}a_{12}^{2}-12B^{2}a_{3}m_{2}a_{12}+6B^{2}a_{1}a_{3}\left(\frac{3}{T}-1\right)+\right.\\ \left.45B^{2}a_{3}^{2}m_{2}\left(\frac{3}{T}-1\right)+3B^{2}m_{2}a_{12}^{2}\left(\frac{3}{T}-1\right)+6B^{2}a_{3}m_{2}a_{12}\left(\frac{3}{T}-1\right)\right)\\ A_{3}=2B\left(6a_{1}\left(\frac{135}{T}-15\right)+18a_{3}m_{2}\left(\frac{135}{T}-15\right)+6m_{2}a_{12}\left(\frac{135}{T}-15\right)\right)-\\ 30B^{2}\left(a_{1}^{2}\left(\frac{135}{T}-15\right)+15a_{3}^{2}m_{4}\left(\frac{135}{T}-15\right)+m_{4}a_{12}^{2}\left(\frac{135}{T}-15\right)+\right.\\ \left.18m_{2}^{2}a_{12}^{2}\left(\frac{3}{T}-1\right)+6a_{1}m_{2}a_{12}\left(\frac{3}{T}-1\right)+6a_{3}m_{4}a_{12}\left(\frac{3}{T}-1\right)+\right.\\ \left.12a_{1}a_{3}m_{2}\left(\frac{135}{T}-15\right)+2a_{1}m_{2}a_{12}\left(\frac{135}{T}-15\right)+12a_{3}m_{2}^{2}a_{12}\left(\frac{135}{T}-15\right)\right)-\\ 105T\left(B^{2}a_{1}^{2}-2B\left(a_{1}+3a_{3}m_{2}+m_{2}a_{12}\right)+\right.\\ \left.15B^{2}a_{3}^{2}m_{4}+B^{2}m_{4}a_{12}^{2}+6B^{2}m_{2}^{2}a_{12}^{2}+12B^{2}a_{1}a_{3}m_{2}+\right.\\ \left.4B^{2}a_{1}m_{2}a_{12}+2B^{2}a_{3}m_{4}a_{12}+12B^{2}a_{3}m_{2}^{2}a_{12}\right)\\ A_{4}=-105T\left(B^{2}a_{1}^{2}m_{2}+2m_{4}B^{2}a_{1}a_{3}+2B^{2}a_{1}m_{2}^{2}a_{12}+\right.\\ \left.m_{6}B^{2}a_{3}^{2}+2m_{4}B^{2}a_{3}m_{2}a_{12}+m_{4}B^{2}m_{2}a_{12}^{2}\right)-\\ 30B^{2}\left(3a_{1}^{2}m_{2}\left(\frac{3}{T}-1\right)+3a_{3}^{2}m_{6}\left(\frac{3}{T}-1\right)+6a_{1}a_{3}m_{4}\left(\frac{3}{T}-1\right)+\right.\\ \left.6a_{1}m_{2}^{2}a_{12}\left(\frac{3}{T}-1\right)+3m_{2}m_{4}a_{12}^{2}\left(\frac{3}{T}-1\right)+6a_{3}m_{2}m_{4}a_{12}\left(\frac{3}{T}-1\right)\right)\\ where\\ m_{l}=E\left[x_{1}^{l}\left[n\right]\right];\;\;l=2,4,6;\;\;B=2m_{2}\mu N\sum_{k=0}^{R-1}{\left|h_{k}\left[n\right]\right|}^{2}\;\;T\gg 100 \end{array}$$

**Proof of Theorem 1.**  Since we deal with the 16QAM constellation input (a two-independent quadrature carrier case), we consider in the following only the pdf of the real part of the convolutional noise. Please note that the pdf of the imaginary part of the convolutional noise is approximately equal to the pdf of the real part of the convolutional noise. The pdf of the real part of the convolutional noise at time indexes *n* and n+1, can be approximately expressed with the help of the Maximum Entropy density approximation technique [1,2,45,46] with Lagrange multipliers up to order six as:

(13) $$\begin{array}{c} f\left(p_{1}\left[n\right]\right)≃\exp\left(\lambda_{0}+\lambda_{2}p_{1}^{2}\left[n\right]+\lambda_{4}p_{1}^{4}\left[n\right]+\lambda_{6}p_{1}^{6}\left[n\right]\right)\\ f\left(p_{1}\left[n+1\right]\right)≃\exp\left(\lambda_{0}+\lambda_{2}p_{1}^{2}\left[n+1\right]+\lambda_{4}p_{1}^{4}\left[n+1\right]+\lambda_{6}p_{1}^{6}\left[n+1\right]\right) \end{array}$$

where $\lambda_{2}$, $\lambda_{4}$ and $\lambda_{6}$ are the Lagrange multipliers up to order six. Next, the difference between the pdf of the real part of the convolutional noise at time index n+1 with that of time index *n* is given by:

(14) $$\begin{array}{c} \Delta f=f\left(p_{1}\left[n+1\right]\right)-f\left(p_{1}\left[n\right]\right)=\\ \exp\left(\lambda_{0}+\lambda_{2}p_{1}^{2}\left[n+1\right]+\lambda_{4}p_{1}^{4}\left[n+1\right]+\lambda_{6}p_{1}^{6}\left[n+1\right]\right)-\exp\left(\lambda_{0}+\lambda_{2}p_{1}^{2}\left[n\right]+\lambda_{4}p_{1}^{4}\left[n\right]+\lambda_{6}p_{1}^{6}\left[n\right]\right)\\ =\exp\left(\lambda_{0}+\lambda_{2}p_{1}^{2}\left[n\right]+\lambda_{4}p_{1}^{4}\left[n\right]+\lambda_{6}p_{1}^{6}\left[n\right]\right)\left(\exp\left(\lambda_{2}\left(p_{1}^{2}\left[n+1\right]-p_{1}^{2}\left[n\right]\right)+\right.\right.\\ \left.\left.\lambda_{4}(p_{1}^{4}\left[n+1\right]-p_{1}^{4}\left[n\right]\right)+\lambda_{6}\left(p_{1}^{6}\left[n+1\right]-p_{1}^{6}\left[n\right]\right)-1\right) \end{array}$$

At the convergence state of the equalizer we may assume that:

(15) Δf≃0

Thus, based on (14) and (15) we may write:

(16) $$E\left[\exp\left(\lambda_{2}\left(p_{1}^{2}\left[n+1\right]-p_{1}^{2}\left[n\right]\right)+\lambda_{4}\left(p_{1}^{4}\left[n+1\right]-p_{1}^{4}\left[n\right]\right)+\lambda_{6}\left(p_{1}^{6}\left[n+1\right]-p_{1}^{6}\left[n\right]\right)\right)-1\right]≃0$$

By using Taylor’s expansion for the exponent [59] (exp(Q)≃1+Q) we may write (16) as:

(17) $$\begin{array}{c} E\left[\exp\left(\lambda_{2}\left(p_{1}^{2}\left[n+1\right]-p_{1}^{2}\left[n\right]\right)+\lambda_{4}\left(p_{1}^{4}\left[n+1\right]-p_{1}^{4}\left[n\right]\right)+\lambda_{6}\left(p_{1}^{6}\left[n+1\right]-p_{1}^{6}\left[n\right]\right)\right)-1\right]≃\\ E\left[\lambda_{2}\left(p_{1}^{2}\left[n+1\right]-p_{1}^{2}\left[n\right]\right)+\lambda_{4}\left(p_{1}^{4}\left[n+1\right]-p_{1}^{4}\left[n\right]\right)+\lambda_{6}\left(p_{1}^{6}\left[n+1\right]-p_{1}^{6}\left[n\right]\right)\right]≃0 \end{array}$$

Based on (17) we have for $E\left[(p_{1}^{6}\left[n+1\right]-p_{1}^{6}\left[n\right])\right]\neq 0$:

(18) $$\begin{array}{c} \lambda_{6}=-\lambda_{2}G-\lambda_{4}F\\ where\\ G=\frac{E\left[p_{1}^{2}\left[n+1\right]-p_{1}^{2}\left[n\right]\right]}{E\left[(p_{1}^{6}\left[n+1\right]-p_{1}^{6}\left[n\right])\right]}\;\;F=\frac{E\left[p_{1}^{4}\left[n+1\right]-p_{1}^{4}\left[n\right]\right]}{E\left[(p_{1}^{6}\left[n+1\right]-p_{1}^{6}\left[n\right])\right]} \end{array}$$

By using (13) and (18) we have:

(19) $$\begin{array}{c} f\left(p_{1}\left[n\right]\right)≃\exp\left(\lambda_{0}+\lambda_{2}p_{1}^{2}\left[n\right]+\lambda_{4}p_{1}^{4}\left[n\right]-\lambda_{2}Gp_{1}^{6}\left[n\right]-\lambda_{4}Fp_{1}^{6}\left[n\right]\right) \end{array}$$

In order to find closed-form approximated expressions for $\lambda_{0}$, $\lambda_{2}$ and $\lambda_{4}$ as a function of the convolutional noise statistics, we use:

(20) $$\begin{array}{c} \int_{-\infty }^{\infty }f\left(p_{1}\left[n\right]\right)=1\\ \int_{-\infty }^{\infty }p_{1}^{2}\left[n\right]f\left(p_{1}\left[n\right]\right)dp_{1}\left[n\right]=E\left[p_{1}^{2}\left[n\right]\right]\\ \int_{-\infty }^{\infty }p_{1}^{4}\left[n\right]f\left(p_{1}\left[n\right]\right)dp_{1}\left[n\right]=E\left[p_{1}^{4}\left[n\right]\right] \end{array}$$

Now, based on (19) and (20) we can write:

(21) $$\begin{array}{c} \int_{-\infty }^{\infty }\exp\left(\lambda_{0}+\lambda_{2}p_{1}^{2}\left[n\right]+\lambda_{4}p_{1}^{4}\left[n\right]-\lambda_{2}Gp_{1}^{6}\left[n\right]-\lambda_{4}Fp_{1}^{6}\left[n\right]\right)dp_{1}\left[n\right]≃1\\ \int_{-\infty }^{\infty }p_{1}^{2}\left[n\right]\exp\left(\lambda_{0}+\lambda_{2}p_{1}^{2}\left[n\right]+\lambda_{4}p_{1}^{4}\left[n\right]-\lambda_{2}Gp_{1}^{6}\left[n\right]-\lambda_{4}Fp_{1}^{6}\left[n\right]\right)dp_{1}\left[n\right]≃E\left[p_{1}^{2}\left[n\right]\right]\\ \int_{-\infty }^{\infty }p_{1}^{4}\left[n\right]\exp\left(\lambda_{0}+\lambda_{2}p_{1}^{2}\left[n\right]+\lambda_{4}p_{1}^{4}\left[n\right]-\lambda_{2}Gp_{1}^{6}\left[n\right]-\lambda_{4}Fp_{1}^{6}\left[n\right]\right)dp_{1}\left[n\right]≃E\left[p_{1}^{4}\left[n\right]\right] \end{array}$$

The first integral in (21) can be written as:

(22) $$\begin{array}{c} \exp\left(\lambda_{0}\right)\int_{-\infty }^{\infty }\exp\left(\lambda_{4}p_{1}^{4}\left[n\right]-\lambda_{2}Gp_{1}^{6}\left[n\right]-\lambda_{4}Fp_{1}^{6}\left[n\right]\right)\exp\left(\lambda_{2}\left(p_{1}^{2}\left[n\right]\right)\right)dp_{1}\left[n\right]=\\ \exp\left(\lambda_{0}\right)\int_{-\infty }^{\infty }g_{1}\left(p_{1}\left[n\right]\right)\exp\left(-\frac{\Psi \left(p_{1}\left[n\right]\right)}{\gamma }\right)dp_{1}\left[n\right]\\ \mathrm{where}\\ g_{1}\left(p_{1}\left[n\right]\right)=\exp\left(\lambda_{4}p_{1}^{4}\left[n\right]-\lambda_{2}Gp_{1}^{6}\left[n\right]-\lambda_{4}Fp_{1}^{6}\left[n\right]\right)\\ \Psi \left(p_{1}\left[n\right]\right)=p_{1}^{2}\left[n\right]\;\;\gamma =-\frac{1}{\lambda_{2}} \end{array}$$

According to the Laplace’s integral method [51], we can solve the integral in (22) by:

(23) $$\begin{array}{c} \int_{-\infty }^{\infty }g_{1}\left(p_{1}\left[n\right]\right)\exp\left(-\frac{\Psi \left(p_{1}\left[n\right]\right)}{\gamma }\right)dp_{1}\left[n\right]≃\\ \exp\left(-\frac{\Psi \left(p_{0}\right)}{\gamma }\right)\sqrt{\frac{2\pi \gamma }{\Psi^{\prime \prime }\left(p_{0}\right)}}\left(g_{1}\left(p_{0}\right)+\frac{g_{1}^{\prime \prime }\left(p_{0}\right)}{2}\frac{\gamma }{\Psi^{\prime \prime }\left(p_{0}\right)}+\frac{g_{1}^{⁗}\left(p_{0}\right)}{8}{\left(\frac{\gamma }{\Psi^{\prime \prime }\left(p_{0}\right)}\right)}^{2}+\right.\\ \left.\frac{g_{1}^{VI}\left(p_{0}\right)}{48}{\left(\frac{\gamma }{\Psi^{\prime \prime }\left(p_{0}\right)}\right)}^{3}+\frac{g_{1}^{VIII}\left(p_{0}\right)}{384}{\left(\frac{\gamma }{\Psi^{\prime \prime }\left(p_{0}\right)}\right)}^{4}+\frac{g_{1}^{X}\left(p_{0}\right)}{3840}{\left(\frac{\gamma }{\Psi^{\prime \prime }\left(p_{0}\right)}\right)}^{5}+O\left(\frac{\gamma^{6}}{{\left(\Psi^{\prime \prime }\left(p_{0}\right)\right)}^{6}}\right)\right) \end{array}$$

where ${\left(\right)}^{\prime \prime }$, ${\left(\right)}^{⁗}$, ${\left(\right)}^{VI}$, ${\left(\right)}^{VIII}$ and ${\left(\right)}^{X}$ denote the second, fourth, sixth, eighth and tenth derivative of (), respectively. $O\left(v\right)$ is defined as $lim_{v\to 0}O\left(v\right)/v=r_{const}$ and $r_{const}$ is a constant. The function $\Psi^{\prime \prime }\left(p_{0}\right)$ and $\left(p_{0}\right)$ are obtained via:

(24) $$\begin{array}{c} \Psi^{\prime }\left(p_{1}\left[n\right]\right)=2p_{1}\left[n\right]⟹\Psi^{\prime \prime }\left(p_{1}\left[n\right]\right)=2\\ \Psi^{\prime }\left(p_{1}\left[n\right]\right)=2p_{1}\left[n\right]⟹\Psi^{\prime }\left(p_{0}\right)=2p_{0}=0⟹p_{0}=0 \end{array}$$

while:

(25) $$\begin{array}{c} g_{1}\left(p_{0}\right)=1;\;\;g_{1}^{\prime \prime }\left(p_{0}\right)=0;\;\;g_{1}^{⁗}\left(p_{0}\right)=24\lambda_{4};\;\;g_{1}^{VI}\left(p_{0}\right)=-720\left(F\lambda_{4}+G\lambda_{2}\right) \end{array}$$

Based on (23)–(25), we may write (22) as:

(26) $$\begin{array}{c} \exp\left(\lambda_{0}\right)\int_{-\infty }^{\infty }\exp\left(\lambda_{4}p_{1}^{4}\left[n\right]-\lambda_{2}Gp_{1}^{6}\left[n\right]-\lambda_{4}Fp_{1}^{6}\left[n\right]\right)\exp\left(\lambda_{2}\left(p_{1}^{2}\left[n\right]\right)\right)dp_{1}\left[n\right]≃\\ \exp(\lambda_{0})\sqrt{\frac{-2\pi \frac{1}{\lambda_{2}}}{2}}\left(1+\frac{24\lambda_{4}}{8}{\left(\frac{-\frac{1}{\lambda_{2}}}{2}\right)}^{2}-720\frac{F\lambda_{4}+G\lambda_{2}}{48}{\left(\frac{-\frac{1}{\lambda_{2}}}{2}\right)}^{3}\right)≃\\ \exp\left(\lambda_{0}\right)\sqrt{\frac{-\pi }{\lambda_{2}}}\left(1+\frac{3\lambda_{4}}{4\lambda_{2}^{2}}+\frac{15\left(F\lambda_{4}+G\lambda_{2}\right)}{8\lambda_{2}^{3}}\right) \end{array}$$

Based on (21) and (26) we have:

(27) $$\exp\left(-\lambda_{0}\right)≃\sqrt{\frac{-\pi }{\lambda_{2}}}\left(1+\frac{3\lambda_{4}}{4\lambda_{2}^{2}}+\frac{15\left(F\lambda_{4}+G\lambda_{2}\right)}{8\lambda_{2}^{3}}\right)$$

Next, we turn to calculate the second integral in (21), which can be expressed as:

(28) $$\begin{array}{c} \exp\left(\lambda_{0}\right)\int_{-\infty }^{\infty }p_{1}^{2}\left[n\right]\exp\left(\lambda_{4}p_{1}^{4}\left[n\right]-\lambda_{2}Gp_{1}^{6}\left[n\right]-\lambda_{4}Fp_{1}^{6}\left[n\right]\right)\exp\left(\lambda_{2}\left(p_{1}^{2}\left[n\right]\right)\right)dp_{1}\left[n\right]=\\ \exp\left(\lambda_{0}\right)\int_{-\infty }^{\infty }g_{2}\left(p_{1}\left[n\right]\right)\exp\left(-\frac{\Psi \left(p_{1}\left[n\right]\right)}{\gamma }\right)dp_{1}\left[n\right]\\ \mathrm{where}\\ g_{2}\left(p_{1}\left[n\right]\right)=p_{1}^{2}\left[n\right]\exp\left(\lambda_{4}p_{1}^{4}\left[n\right]-\lambda_{2}Gp_{1}^{6}\left[n\right]-\lambda_{4}Fp_{1}^{6}\left[n\right]\right) \end{array}$$

According to Laplace’s integral method [51], we can write (28) as:

(29) $$\begin{array}{c} \int_{-\infty }^{\infty }g_{2}\left(p_{1}\left[n\right]\right)\exp\left(-\frac{\Psi \left(p_{1}\left[n\right]\right)}{\gamma }\right)dp_{1}\left[n\right]≃\\ \exp\left(-\frac{\Psi \left(p_{0}\right)}{\gamma }\right)\sqrt{\frac{2\pi \gamma }{\Psi^{\prime \prime }\left(p_{0}\right)}}\left(g_{2}\left(p_{0}\right)+\frac{g_{2}^{\prime \prime }\left(p_{0}\right)}{2}\frac{\gamma }{\Psi^{\prime \prime }\left(p_{0}\right)}+\frac{g_{2}^{⁗}\left(p_{0}\right)}{8}{\left(\frac{\gamma }{\Psi^{\prime \prime }\left(p_{0}\right)}\right)}^{2}+\right.\\ \left.\frac{g_{2}^{VI}\left(p_{0}\right)}{48}{\left(\frac{\gamma }{\Psi^{\prime \prime }\left(p_{0}\right)}\right)}^{3}+\frac{g_{2}^{VIII}\left(p_{0}\right)}{384}{\left(\frac{\gamma }{\Psi^{\prime \prime }\left(p_{0}\right)}\right)}^{4}+O\left(\frac{\gamma^{5}}{{\left(\Psi^{\prime \prime }\left(p_{0}\right)\right)}^{5}}\right)\right) \end{array}$$

where:

(30) $$\begin{array}{c} g_{2}\left(p_{0}\right)=0;\;\;g_{2}^{\prime \prime }\left(p_{0}\right)=2;\;\;g_{2}^{⁗}\left(p_{0}\right)=0\\ g_{2}^{VI}\left(p_{0}\right)=720\lambda_{4};\;\;g_{2}^{VIII}\left(p_{0}\right)=-40320F\lambda_{4}-40320G\lambda_{2} \end{array}$$

Based on (21), (29) and (30) we may write (28) as:

(31) $$\begin{array}{c} \exp\left(\lambda_{0}\right)\int_{-\infty }^{\infty }p_{1}^{2}\left[n\right]\exp\left(\lambda_{4}p_{1}^{4}\left[n\right]-\lambda_{2}Gp_{1}^{6}\left[n\right]-\lambda_{4}Fp_{1}^{6}\left[n\right]\right)\exp\left(\lambda_{2}\left(p_{1}^{2}\left[n\right]\right)\right)dp_{1}\left[n\right]≃\\ \exp\left(\lambda_{0}\right)\sqrt{\frac{-\pi }{\lambda_{2}}}\left(-\frac{1}{2\lambda_{2}}-15\lambda_{4}\frac{1}{8\lambda_{2}^{3}}-\left(105F\lambda_{4}+105G\lambda_{2}\right)\frac{1}{16\lambda_{2}^{4}}\right)≃m_{p} \end{array}$$

Based on (27) and (31) we have:

(32) $$\begin{array}{c} \sqrt{\frac{-\pi }{\lambda_{2}}}\left(-\frac{1}{2\lambda_{2}}-15\lambda_{4}\frac{1}{8\lambda_{2}^{3}}-\left(105F\lambda_{4}+105G\lambda_{2}\right)\frac{1}{16\lambda_{2}^{4}}\right)≃\\ m_{p}\exp\left(-\lambda_{0}\right)≃m_{p}\sqrt{\frac{-\pi }{\lambda_{2}}}\left(1+\frac{3\lambda_{4}}{4\lambda_{2}^{2}}+\frac{15\left(F\lambda_{4}+G\lambda_{2}\right)}{8\lambda_{2}^{3}}\right)\\ ⇓\\ \lambda_{4}≃\frac{\left(-\frac{1}{2\lambda_{2}}-105G\lambda_{2}\frac{1}{16\lambda_{2}^{4}}\right)-m_{p}\left(1+\frac{15G\lambda_{2}}{8\lambda_{2}^{3}}\right)}{\frac{15}{8\lambda_{2}^{3}}+\frac{105F}{16\lambda_{2}^{4}}+m_{p}\left(\frac{3}{4\lambda_{2}^{2}}+\frac{15F}{8\lambda_{2}^{3}}\right)} \end{array}$$

Next, we turn to calculate the third integral in (21), which can be expressed as:

(33) $$\begin{array}{c} \exp\left(\lambda_{0}\right)\int_{-\infty }^{\infty }p_{1}^{4}\left[n\right]\exp\left(\lambda_{4}p_{1}^{4}\left[n\right]-\lambda_{2}Gp_{1}^{6}\left[n\right]-\lambda_{4}Fp_{1}^{6}\left[n\right]\right)\exp\left(\lambda_{2}\left(p_{1}^{2}\left[n\right]\right)\right)dp_{1}\left[n\right]=\\ \exp\left(\lambda_{0}\right)\int_{-\infty }^{\infty }g_{3}\left(p_{1}\left[n\right]\right)\exp\left(-\frac{\Psi \left(p_{1}\left[n\right]\right)}{\gamma }\right)dp_{1}\left[n\right]\\ \mathrm{where}\\ g_{3}\left(p_{1}\left[n\right]\right)=p_{1}^{4}\left[n\right]\exp\left(\lambda_{4}p_{1}^{4}\left[n\right]-\lambda_{2}Gp_{1}^{6}\left[n\right]-\lambda_{4}Fp_{1}^{6}\left[n\right]\right) \end{array}$$

According to Laplace’s integral method [51], we can write (33) as:

(34) $$\begin{array}{c} \int_{-\infty }^{\infty }g_{3}\left(p_{1}\left[n\right]\right)\exp\left(-\frac{\Psi \left(p_{1}\left[n\right]\right)}{\gamma }\right)dp_{1}\left[n\right]≃\\ \exp\left(-\frac{\Psi \left(p_{0}\right)}{\gamma }\right)\sqrt{\frac{2\pi \gamma }{\Psi^{\prime \prime }\left(p_{0}\right)}}\left(g_{3}\left(p_{0}\right)+\frac{g_{3}^{\prime \prime }\left(p_{0}\right)}{2}\frac{\gamma }{\Psi^{\prime \prime }\left(p_{0}\right)}+\frac{g_{3}^{⁗}\left(p_{0}\right)}{8}{\left(\frac{\gamma }{\Psi^{\prime \prime }\left(p_{0}\right)}\right)}^{2}+\right.\\ \left.\frac{g_{3}^{VI}\left(p_{0}\right)}{48}{\left(\frac{\gamma }{\Psi^{\prime \prime }\left(p_{0}\right)}\right)}^{3}+\frac{g_{3}^{VIII}\left(p_{0}\right)}{384}{\left(\frac{\gamma }{\Psi^{\prime \prime }\left(p_{0}\right)}\right)}^{4}+\frac{g_{3}^{X}\left(p_{0}\right)}{3840}{\left(\frac{\gamma }{\Psi^{\prime \prime }\left(p_{0}\right)}\right)}^{5}+O\left(\frac{\gamma^{6}}{{\left(\Psi^{\prime \prime }\left(p_{0}\right)\right)}^{6}}\right)\right) \end{array}$$

where:

(35) $$\begin{array}{c} g_{3}\left(p_{0}\right)=0;\;\;g_{3}^{\prime \prime }\left(p_{0}\right)=0;\;\;g_{3}^{⁗}\left(p_{0}\right)=24\\ g_{3}^{VI}\left(p_{0}\right)=0;\;\;g_{3}^{VIII}\left(p_{0}\right)=40320\lambda_{4};\;\;g_{3}^{X}\left(p_{0}\right)=-3628800F\lambda_{4}-3628800G\lambda_{2} \end{array}$$

Based on (21), (34) and (35) we can write (33) as:

(36) $$\begin{array}{c} \exp\left(\lambda_{0}\right)\int_{-\infty }^{\infty }p_{1}^{4}\left[n\right]\exp\left(\lambda_{4}p_{1}^{4}\left[n\right]-\lambda_{2}Gp_{1}^{6}\left[n\right]-\lambda_{4}Fp_{1}^{6}\left[n\right]\right)\exp\left(\lambda_{2}\left(p_{1}^{2}\left[n\right]\right)\right)dp_{1}\left[n\right]≃\\ \exp\left(\lambda_{0}\right)\sqrt{\frac{-\pi }{\lambda_{2}}}\left(3\left(\frac{1}{4\lambda_{2}^{2}}\right)+105\lambda_{4}\left(\frac{1}{16\lambda_{2}^{4}}\right)-945\left(F\lambda_{4}+G\lambda_{2}\right)\left(\frac{1}{32\lambda_{2}^{5}}\right)\right)=E\left[p_{1}^{4}\left[n\right]\right] \end{array}$$

Based on (27) and (36) we may write:

(37) $$\begin{array}{c} \sqrt{\frac{-\pi }{\lambda_{2}}}\left(3\left(\frac{1}{4\lambda_{2}^{2}}\right)+105\lambda_{4}\left(\frac{1}{16\lambda_{2}^{4}}\right)-945\left(F\lambda_{4}+G\lambda_{2}\right)\left(\frac{1}{32\lambda_{2}^{5}}\right)\right)≃\\ \sqrt{\frac{-\pi }{\lambda_{2}}}\left(1+\frac{3\lambda_{4}}{4\lambda_{2}^{2}}+\frac{15\left(F\lambda_{4}+G\lambda_{2}\right)}{8\lambda_{2}^{3}}\right)E\left[p_{1}^{4}\left[n\right]\right]\\ ⇓\\ \lambda_{4}≃\frac{\left(1+\frac{15G}{8\lambda_{2}^{2}}\right)E\left[p_{1}^{4}\left[n\right]\right]-\frac{3}{4\lambda_{2}^{2}}+945G\left(\frac{1}{32\lambda_{2}^{4}}\right)}{\frac{105}{16\lambda_{2}^{4}}-945F\frac{1}{32\lambda_{2}^{5}}-\frac{3}{4\lambda_{2}^{2}}-\frac{15F}{8\lambda_{2}^{3}}} \end{array}$$

However, we already received $\lambda_{4}$ in (32). Thus, the expression for $\lambda_{4}$ in (37) and that obtained in (32) should be approximately the same. Thus, we may write:

(38) $$\begin{array}{c} \frac{\left(-\frac{1}{2\lambda_{2}}-105G\lambda_{2}\frac{1}{16\lambda_{2}^{4}}\right)-m_{p}\left(1+\frac{15G\lambda_{2}}{8\lambda_{2}^{3}}\right)}{\frac{15}{8\lambda_{2}^{3}}+\frac{105F}{16\lambda_{2}^{4}}+m_{p}\left(\frac{3}{4\lambda_{2}^{2}}+\frac{15F}{8\lambda_{2}^{3}}\right)}≃\\ \frac{\left(1+\frac{15G}{8\lambda_{2}^{2}}\right)E\left[p_{1}^{4}\left[n\right]\right]-\frac{3}{4\lambda_{2}^{2}}+945G\left(\frac{1}{32\lambda_{2}^{4}}\right)}{\frac{105}{16\lambda_{2}^{4}}-945F\frac{1}{32\lambda_{2}^{5}}-\frac{3}{4\lambda_{2}^{2}}-\frac{15F}{8\lambda_{2}^{3}}} \end{array}$$

Now, for $\frac{8\lambda_{2}^{2}}{105}\gg G$ we may write (38) as:

(39) $$\begin{array}{c} \frac{-\frac{1}{2\lambda_{2}}-m_{p}}{\frac{15}{8\lambda_{2}^{3}}+\frac{105F}{16\lambda_{2}^{4}}+m_{p}\left(\frac{3}{4\lambda_{2}^{2}}+\frac{15F}{8\lambda_{2}^{3}}\right)}≃\frac{E\left[p_{1}^{4}\left[n\right]\right]-\frac{3}{4\lambda_{2}^{2}}+945G\left(\frac{1}{32\lambda_{2}^{4}}\right)}{\frac{105}{16\lambda_{2}^{4}}-945F\frac{1}{32\lambda_{2}^{5}}-\frac{3}{4\lambda_{2}^{2}}-\frac{15F}{8\lambda_{2}^{3}}} \end{array}$$

Next, let us write $G≃\frac{8\lambda_{2}^{2}}{105T}$ where *T* is very large positive value and use it in (39):

(40) $$\begin{array}{c} \frac{-\frac{1}{2\lambda_{2}}-m_{p}}{\frac{15}{8\lambda_{2}^{3}}+\frac{105F}{16\lambda_{2}^{4}}+m_{p}\left(\frac{3}{4\lambda_{2}^{2}}+\frac{15F}{8\lambda_{2}^{3}}\right)}≃\frac{E\left[p_{1}^{4}\left[n\right]\right]-\frac{3}{4\lambda_{2}^{2}}+\frac{9}{4\lambda_{2}^{2}T}}{\frac{105}{16\lambda_{2}^{4}}-945F\frac{1}{32\lambda_{2}^{5}}-\frac{3}{4\lambda_{2}^{2}}-\frac{15F}{8\lambda_{2}^{3}}} \end{array}$$

A possible solution for the left side of (40) being equal to its right side is when:

(41) $$\begin{array}{c} -\frac{1}{2\lambda_{2}}≃m_{p};\;\;E\left[p_{1}^{4}\left[n\right]\right]-\frac{3}{4\lambda_{2}^{2}}+\frac{9}{4\lambda_{2}^{2}T}≃0\\ ⇓\\ m_{p}≃-\frac{1}{2\lambda_{2}}\;\Rightarrow \;E\left[p_{1}^{4}\left[n\right]\right]≃3m_{p}^{2}\left(1-\frac{3}{T}\right) \end{array}$$

Please note that when (41) holds it means that $\lambda_{4}≃0$. In addition, for T→∞, $E\left[p_{1}^{4}\left[n\right]\right]\to 3m_{p}^{2}$, which holds in the Gaussian case. Next, by using (18), $G≃\frac{8\lambda_{2}^{2}}{105T}$ and (41) we may write:

(42) $$\begin{array}{c} \frac{E\left[p^{2}\left[n+1\right]-p^{2}\left[n\right]\right]}{E\left[(p^{6}\left[n+1\right]-p^{6}\left[n\right])\right]}≃\frac{8\lambda_{2}^{2}}{105T}≃\frac{8{\left(-\frac{1}{2m_{p}}\right)}^{2}}{105T}≃\frac{2}{105m_{p}^{2}T}\\ ⇓\\ \lambda_{6}=-\lambda_{2}\frac{E\left[p_{1}^{2}\left[n+1\right]-p_{1}^{2}\left[n\right]\right]}{E\left[(p_{1}^{6}\left[n+1\right]-p_{1}^{6}\left[n\right])\right]}-\lambda_{4}\frac{E\left[p_{1}^{4}\left[n+1\right]-p_{1}^{4}\left[n\right]\right]}{E\left[(p_{1}^{6}\left[n+1\right]-p_{1}^{6}\left[n\right])\right]}≃\\ -\lambda_{2}\frac{E\left[p_{1}^{2}\left[n+1\right]-p_{1}^{2}\left[n\right]\right]}{E\left[(p_{1}^{6}\left[n+1\right]-p_{1}^{6}\left[n\right])\right]}≃\frac{1}{105m_{p}^{3}T} \end{array}$$

By using (27), $G≃\frac{8\lambda_{2}^{2}}{105T}$, (41) and (42) we may write $f\left(p_{1}\left[n\right]\right)$ in (13) as:

(43) $$\begin{array}{c} f\left(p_{1}\left[n\right]\right)≃\exp\left(\lambda_{0}+\lambda_{2}p_{1}^{2}\left[n\right]+\lambda_{4}p_{1}^{4}\left[n\right]+\lambda_{6}p_{1}^{6}\left[n\right]\right)≃\\ \frac{1}{\sqrt{2\pi m_{p}}\left(1+\frac{1}{7T}\right)}\exp\left(-\frac{1}{2m_{p}}p_{1}^{2}\left[n\right]+\frac{1}{105m_{p}^{3}T}p_{1}^{6}\left[n\right]\right) \end{array}$$

Please note that for $m_{p}\neq 0$ and T→∞, the convolutional noise pdf given in (43) tends to the Gaussian one. However, this does not tell us for which residual ISI this occurs. Thus, we turn to the expression of $G≃\frac{8\lambda_{2}^{2}}{105T}$ and ask ourselves what is the residual ISI or $m_{p}$ for which $G≃\frac{8\lambda_{2}^{2}}{105T}$ holds. In order to do this, we first have to obtain closed-form expressions for $E\left[p^{2}\left[n+1\right]-p^{2}\left[n\right]\right]$ and $E\left[p^{6}\left[n+1\right]-p^{6}\left[n\right]\right]$. According to [58], we have:

(44) $$\begin{array}{c} \Delta {\tilde{g}}_{i}=\frac{\partial {\tilde{g}}_{i}}{\partial p_{1}\left[n\right]}\Delta p_{1}+\frac{1}{2}\frac{\partial^{2}{\tilde{g}}_{i}}{\partial^{2}p_{1}\left[n\right]}{\left(\Delta p_{1}\right)}^{2}+O{\left(\Delta p_{1}\right)}^{3};\;\;i=1,2;\\ \mathrm{where}\\ \Delta {\tilde{g}}_{i}={\tilde{g}}_{i}\left[n+1\right]-{\tilde{g}}_{i}\left[n\right];\;\;\Delta p_{1}=-Re\left[\mu P\left[z\left[n\right]\right]\sum_{m=0}^{m=R-1}y\left[n-m\right]y^{*}\left[n-m\right]\right] \end{array}$$

where P[z[n]] and Re[P[z[n]]] are given in (9) and (10) respectively. Thus, based on (44) we have:

(45) $$\begin{array}{c} {\tilde{g}}_{1}\left[n\right]=p_{1}^{2}\left[n\right]\;\Rightarrow \;E\left[p_{1}^{2}\left[n+1\right]-p_{1}^{2}\left[n\right]\right]≃2E\left[p_{1}\left[n\right]\Delta p_{1}\right]+E\left[{\left(\Delta p_{1}\right)}^{2}\right]\\ {\tilde{g}}_{2}\left[n\right]=p_{1}^{6}\left[n\right]\;\Rightarrow \;E\left[p_{1}^{6}\left[n+1\right]-p_{1}^{6}\left[n\right]\right]≃6E\left[p_{1}^{5}\left[n\right]\Delta p_{1}\right]+15E\left[p_{1}^{4}\left[n\right]{\left(\Delta p_{1}\right)}^{2}\right] \end{array}$$

In the following, we assume that $E\left[{\left(\sum_{m=0}^{m=R-1}y\left[n-m\right]y^{*}\left[n-m\right]\right)}^{2}\right]≃B^{2}$, as was done in [58]. Please note that in [58], the expression for $E\left[p_{1}^{2}\left[n+1\right]-p_{1}^{2}\left[n\right]\right]$ was already derived. However, in [58], the Gaussian case was assumed, while here, we do not use this assumption. Therefore, in our case, all the higher moments (higher than four) associated with the real part of the convolutional noise have to be obtained differently than in [58]. In addition, the obtained expression for $E\left[p_{1}^{2}\left[n+1\right]-p_{1}^{2}\left[n\right]\right]$ in [58] was set to zero to find the residual ISI applicable in the convergence state, while in our case it is not set to zero. In order to carry out the calculations of $E\left[p_{1}^{2}\left[n+1\right]-p_{1}^{2}\left[n\right]\right]$ and $E\left[p_{1}^{6}\left[n+1\right]-p_{1}^{6}\left[n\right]\right]$, we need the moments up to order ten of the real part of the convolutional noise. Since we can not use the Gaussian assumption but have on hand the fourth moment of the real part of the convolutional noise (41), thus we only need to find a technique that supplies us with all the higher order moments (higher than four) of the real part of the convolutional noise. The quasi moment truncation technique is related to the Hermite polynomials where the high-order central moments are approximated in terms of lower order central moments [48]. Thus, according to the quasi moment truncation technique [48] and with the help of (41) we have:

(46) $$\begin{array}{c} E\left[p_{1}^{6}\right]≃15m_{p}E\left[p_{1}^{4}\right]-30m_{p}^{3}=\left(15-\frac{135}{T}\right)m_{p}^{3}\\ E\left[p_{1}^{8}\right]≃28m_{p}E\left[p_{1}^{6}\right]-210m_{p}^{2}E\left[p_{1}^{4}\right]+315m_{p}^{4}=\left(105-\frac{1890}{T}\right)m_{p}^{4}\\ E\left[p_{1}^{10}\right]≃45m_{p}E\left[p_{1}^{8}\right]-630m_{p}^{2}E\left[p_{1}^{6}\right]+3150m_{p}^{3}E\left[p_{1}^{4}\right]-3780m_{p}^{5}=\left(945-\frac{28\;350}{T}\right)m_{p}^{5} \end{array}$$

Next, by using (41), (44)–(46), we may write:

(47) $$\begin{array}{c} E\left[p_{1}^{2}\left[n+1\right]-p_{1}^{2}\left[n\right]\right]≃\left(-3B^{2}a_{12}^{2}\left(\frac{3}{T}-1\right)-B^{2}a_{3}^{2}\left(\frac{135}{T}-15\right)-\right.\\ \left.6B^{2}a_{3}a_{12}\left(\frac{3}{T}-1\right)\right)m_{p}^{3}+\left(2B^{2}a_{1}a_{12}-2B\left(a_{12}-3a_{3}\left(\frac{3}{T}-1\right)\right)+\right.\\ \left.6B^{2}m_{2}a_{12}^{2}+12B^{2}a_{3}m_{2}a_{12}-6B^{2}a_{1}a_{3}\left(\frac{3}{T}-1\right)-45B^{2}a_{3}^{2}m_{2}\left(\frac{3}{T}-1\right)-\right.\\ \left.3B^{2}m_{2}a_{12}^{2}\left(\frac{3}{T}-1\right)-6B^{2}a_{3}m_{2}a_{12}\left(\frac{3}{T}-1\right)\right)m_{p}^{2}+\\ \left(B^{2}a_{1}^{2}-2B\left(a_{1}+3a_{3}m_{2}+m_{2}a_{12}\right)+15B^{2}a_{3}^{2}m_{4}+B^{2}m_{4}a_{12}^{2}+6B^{2}m_{2}^{2}a_{12}^{2}+\right.\\ \left.12B^{2}a_{1}a_{3}m_{2}+4B^{2}a_{1}m_{2}a_{12}+2B^{2}a_{3}m_{4}a_{12}+12B^{2}a_{3}m_{2}^{2}a_{12}\right)m_{p}+\\ \left(B^{2}a_{1}^{2}m_{2}+2m_{4}B^{2}a_{1}a_{3}+2B^{2}a_{1}m_{2}^{2}a_{12}+m_{6}B^{2}a_{3}^{2}+\right.\\ \left.2m_{4}B^{2}a_{3}m_{2}a_{12}+m_{4}B^{2}m_{2}a_{12}^{2}\right) \end{array}$$

and:

(48) $$\begin{array}{c} E\left[p_{1}^{6}\left[n+1\right]-p_{1}^{6}\left[n\right]\right]≃\left(-15B^{2}\left(a_{3}^{2}\left(\frac{28\;350}{T}-945\right)-3a_{12}^{2}\left(\frac{3}{T}-1\right)\left(\frac{135}{T}-15\right)+\right.\right.\\ \left.\left.2a_{3}a_{12}\left(\frac{1890}{T}-105\right)\right)\right)m_{p}^{5}+\left(6a_{12}\left(\frac{135}{T}-15\right)-\right.\\ \left.15B^{2}\left(6m_{2}a_{12}^{2}\left(\frac{135}{T}-15\right)+15a_{3}^{2}m_{2}\left(\frac{1890}{T}-105\right)-9m_{2}a_{12}^{2}{\left(\frac{3}{T}-1\right)}^{2}+\right.\right.\\ \left.\left.2a_{1}a_{12}\left(\frac{135}{T}-15\right)+2a_{1}a_{3}\left(\frac{1890}{T}-105\right)+12a_{3}m_{2}a_{12}\left(\frac{135}{T}-15\right)+\right.\right.\\ \left.\left.2a_{3}m_{2}a_{12}\left(\frac{1890}{T}-105\right)\right)+6a_{3}\left(\frac{1890}{T}-105\right)\right)m_{p}^{4}+\\ \left(B\left(6a_{1}\left(\frac{135}{T}-15\right)+18a_{3}m_{2}\left(\frac{135}{T}-15\right)+6m_{2}a_{12}\left(\frac{135}{T}-15\right)\right)-\right.\\ \left.15B^{2}\left(a_{1}^{2}\left(\frac{135}{T}-15\right)+15a_{3}^{2}m_{4}\left(\frac{135}{T}-15\right)+m_{4}a_{12}^{2}\left(\frac{135}{T}-15\right)+\right.\right.\\ \left.\left.18m_{2}^{2}a_{12}^{2}\left(\frac{3}{T}-1\right)+6a_{1}m_{2}a_{12}\left(\frac{3}{T}-1\right)+6a_{3}m_{4}a_{12}\left(\frac{3}{T}-1\right)+12a_{1}a_{3}m_{2}\left(\frac{135}{T}-15\right)+\right.\right.\\ \left.\left.2a_{1}m_{2}a_{12}\left(\frac{135}{T}-15\right)+12a_{3}m_{2}^{2}a_{12}\left(\frac{135}{T}-15\right)\right)\right)m_{p}^{3}+\\ \left(-15B^{2}\left(3a_{1}^{2}m_{2}\left(\frac{3}{T}-1\right)+3a_{3}^{2}m_{6}\left(\frac{3}{T}-1\right)+6a_{1}a_{3}m_{4}\left(\frac{3}{T}-1\right)+\right.\right.\\ \left.\left.6a_{1}m_{2}^{2}a_{12}\left(\frac{3}{T}-1\right)+3m_{2}m_{4}a_{12}^{2}\left(\frac{3}{T}-1\right)+6a_{3}m_{2}m_{4}a_{12}\left(\frac{3}{T}-1\right)\right)\right)m_{p}^{2} \end{array}$$

Based on (42) we may write:

(49) $$2\left(E\left[(p^{6}\left[n+1\right]-p^{6}\left[n\right])\right]\right)-\left(E\left[p^{2}\left[n+1\right]-p^{2}\left[n\right]\right]\right)105m_{p}^{2}T≃0$$

Next, by substituting (47) and (48) into (49) and dividing both sides of the obtained equation by $m_{p}^{2}$ for $m_{p}\neq 0$, we obtain (12). By substituting the solution for $m_{p}$ obtained in (12) into (5), together with $E[x_{1}^{2}\left[n\right]]$, (11) is obtained. □

## 4. Simulation

In the previous section we derived a closed-form approximated expression for the residual ISI (11) as a function of the system’s parameter (step-size parameter, input constellation statistics, equalizer’s tap length, channel power and properties of the chosen equalizer via $a_{1}$, $a_{3}$ and $a_{12}$) for which the pdf of the real part of the convolutional noise can be approximately considered as a Gaussian pdf. In this section we wish to calculate the residual ISI (11) and see if the obtained level for the residual ISI (11) is above the −16 [dB] where the “eye diagram” is still closed. Specifically, when decisions on the equalized output sequence cannot be made with confidence. In the following, we use the channel proposed by [7]: $h_{n}=\left\{0\;\mathrm{for}\;n<0;\;-0.4\;\mathrm{for}\;n=0;\;\;0.84\cdot 0.4^{n-1}\;\mathrm{for}\;n>0\right\}$.

**Figure 2:**
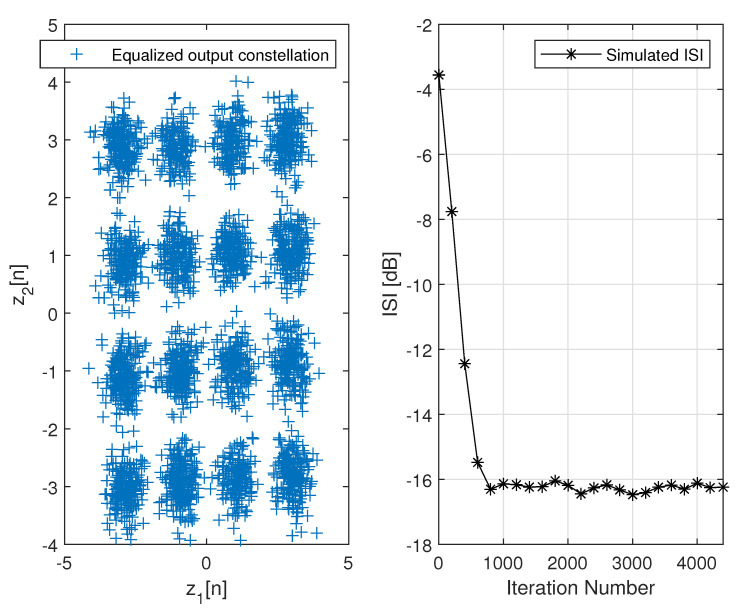
Right side plot: Simulated ISI with Godard’s algorithm for the 16QAM constellation input. For the noiseless case, 100 Monte Carlo runs produced the averaged results. The equalizer’s and channel’s tap length were set to 13 (N=R=13), μ=0.0001. Left side plot: Equalized output constellation with Godard’s algorithm for N=R=13 and μ=0.0001.

**Figure 3:**
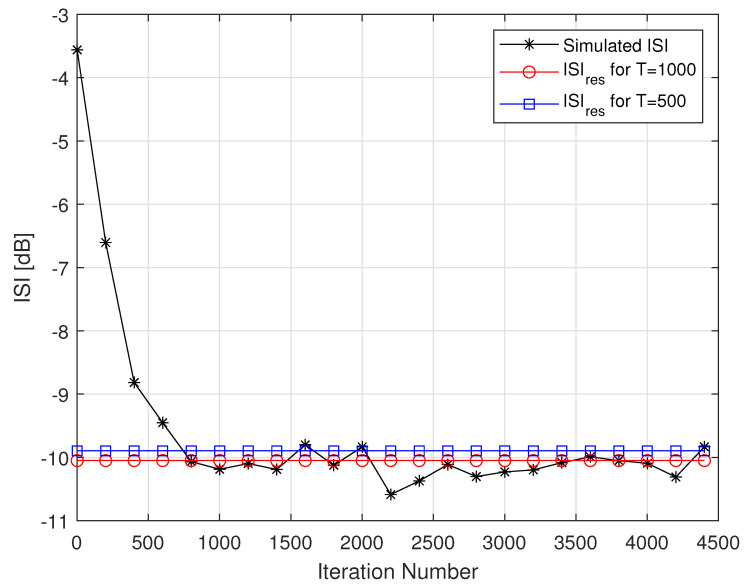
Simulated ISI with Godard’s algorithm for the 16QAM constellation input. For the noiseless case, 100 Monte Carlo runs produced the averaged results. The averaged results were compared with the calculated residual ISI given in (11) for N=R=13, μ=0.00022 and two cases for *T*.

**Figure 4:**
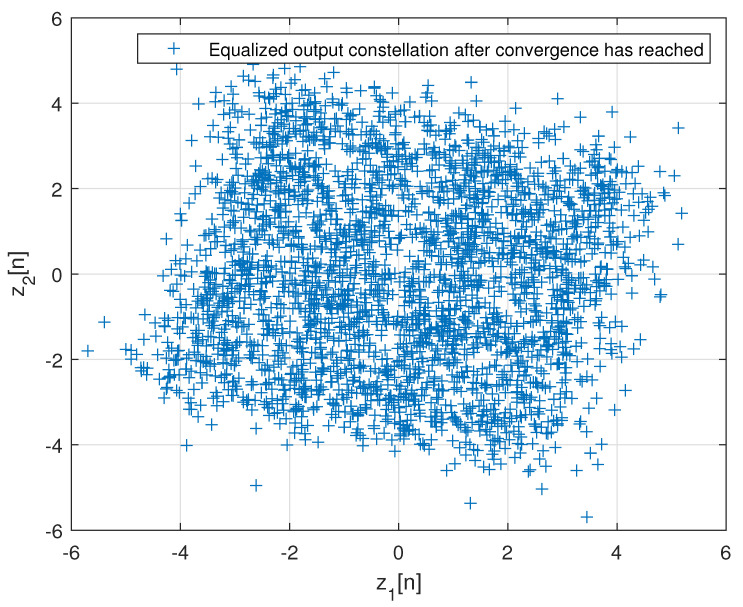
Equalized output constellation with Godard’s algorithm for N=R=13 and μ=0.00022.

**Figure 5:**
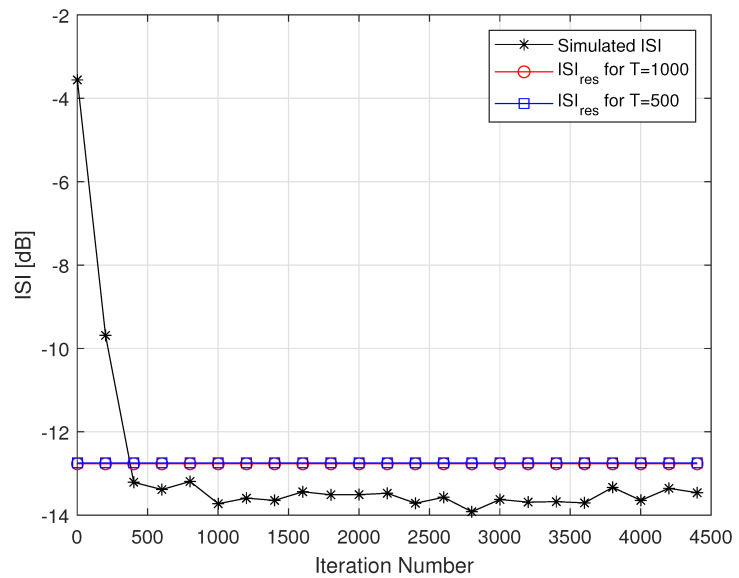
Simulated ISI with the MMA algorithm for the 16QAM constellation input. For the noiseless case, 100 Monte Carlo runs produced the averaged results. The averaged results were compared with the calculated residual ISI given in (11) for N=R=13, μ=0.000365 and two cases for *T*.

**Figure 6:**
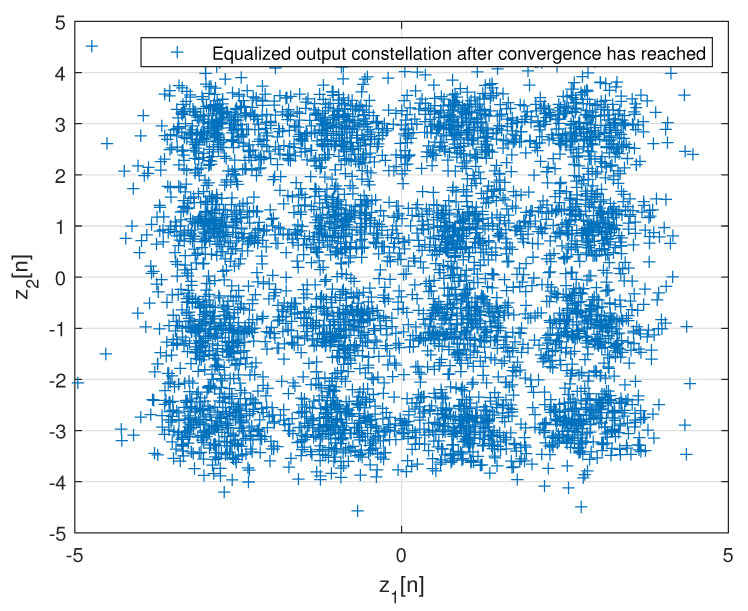
Equalized output constellation with the MMA algorithm for N=R=13 and μ=0.000365.

Figure 2 describes the averaged ISI and equalized output constellation obtained for Godard’s algorithm [52], where decisions on the equalized output sequence can be made rather reliably. Figure 3 describes the comparison between the simulated ISI with Godard’s algorithm [52] and with those calculated via the expression for the residual ISI given in (11) for two different values for *T* and using the values for $a_{1}$, $a_{12}$ and $a_{3}$ associated with Godard’s algorithm [52]. According to Figure 3, the simulated ISI is above the −16 [dB] where decisions on the equalized output sequence cannot be made in a reliable manner as it can be clearly seen from Figure 4. According to Figure 3, the calculated residual ISI (11) for both cases (T=500 and T=1000) is above the obtained level for the simulated ISI. Thus, we have shown here via simulation that the Gaussian assumption for the convolutional noise pdf can be approximately made just before the equalizer has converged, even at a residual ISI level where no trustworthy judgements can be made. Next, we apply the MMA algorithm [55,56] for the blind adaptive equalization task. Figure 5 describes the comparison between the simulated ISI with the MMA algorithm [55,56] and with those calculated via the expression for the residual ISI given in (11) for two different values for *T* and using the values for $a_{1}$, $a_{12}$ and $a_{3}$ associated with the MMA algorithm [55,56]. According to Figure 5, the simulated ISI is above the −16 [dB] where decisions on the equalized output sequence cannot be made in a reliable manner as it can be clearly seen from Figure 6. According to Figure 5, the calculated residual ISI (11) for both cases (T=500 and T=1000) is above the obtained level for the simulated ISI. Thus, also here, we see via simulation that the Gaussian assumption for the convolutional noise pdf can be approximately made just before the equalizer has converged even at a residual ISI level where no reliable decisions can be carried out. Please note that we used T=500 and T=1000 since according to (12) the value for *T* should be T≫100.

## 5. Discussion

In this study, a closed-form approximated expression was established for the residual ISI (11) for which the real part of the convolutional noise’s pdf can be approximately regarded as Gaussian. In the previous section, we have shown via simulation that the Gaussian assumption for the real part of the convolutional noise’s pdf can be approximately made just before the equalizer has converged even at a residual ISI level where no reliable decisions can be made unlike it was believed in the literature [41]. Thus, this may be the reason for achieving satisfactory equalization results from an ISI and acquisition perspective from those blind adaptive equalizer’s ([1,2,6,34,40,41,42,43]) based on the Gaussian assumption throughout the entire deconvolution procedure for the convolutional noise pdf.

Let us go back for a moment to the obtained expression for the pdf associated with the real part of the convolutional noise given in (43). As it was already stated, for $m_{p}\neq 0$ and T→∞, the convolutional noise pdf given in (43) tends to the Gaussian one. Since $G≃\frac{8\lambda_{2}^{2}}{105T}$, it means that G≃0 for T→∞. Now, based on (18), G≃0 for $E\left[p_{1}^{2}\left[n+1\right]-p_{1}^{2}\left[n\right]\right]≃0$. In [58], the expression for $E\left[p_{1}^{2}\left[n+1\right]-p_{1}^{2}\left[n\right]\right]$ was obtained for the Gaussian case and set to zero to find the residual ISI applicable in the convergence state for the noiseless case. Thus, for T→∞, the obtained expression for the residual ISI (11) for which the real part of the convolutional noise’s pdf can be approximately regarded as Gaussian is approximately the obtained expression for the residual ISI applicable in the convergence state given in [58]. This may be the reason why having very satisfying results in [58] for the 16QAM case, even for residual ISI levels above −16 [dB]. Although we considered in this paper only the 16QAM constellation input, the expression for the residual ISI given in (11) holds also for the real valued input case and for any other input constellation that belongs to the two-independent quadrature carrier case as the 64QAM and 256QAM inputs. Although we used only one channel for the simulation task, the expression for the residual ISI given in (11) holds also for any channel that complies with assumption two from the system description section. As was already pointed out, for T→∞, the obtained expression for the residual ISI (11) for which the real part of the convolutional noise’s pdf can be approximately regarded as Gaussian is approximately the obtained expression for the residual ISI applicable in the convergence state given in [58]. Eight different channels and three different input sources (16QAM, 64QAM and 4QAM) were considered in [58] for the simulation task. According to [58], very satisfying simulation results were obtained. Thus, in this paper, there was no need to take more channels and different input sources than the 16QAM for the simulation task.

## Data Availability

All the relevant data is given in the article.
